# Proteomic analysis of a clavata-like phenotype mutant in *Brassica napus*

**DOI:** 10.1590/1678-4685-GMB-2019-0305

**Published:** 2020-03-06

**Authors:** Keming Zhu, Weiwei Zhang, Rehman Sarwa, Shuo Xu, Kaixia Li, Yanhua Yang, Yulong Li, Zheng Wang, Jun Cao, Yaoming Li, Xiaoli Tan

**Affiliations:** 1 Jiangsu University, Institute of Life Sciences, Zhenjiang, Jiangsu, China.; 2 Ministry of Agriculture, Key Laboratory of Biology and Genetic Improvement of Oil Crops, Wuhan, China.; 3 Jiangsu University, Institute of Agricultural Engineering, Zhenjiang, China.

**Keywords:** Brassica napus, proteomic, inflorescence meristem (IM), Bnclavata-like (Bnclv-like), quantitative real-time PCR

## Abstract

Rapeseed is one of important oil crops in China. Better understanding of the regulation network of main agronomic traits of rapeseed could improve the yielding of rapeseed. In this study, we obtained an influrescence mutant that showed a fusion phenotype, similar with the *Arabidopsis* clavata-like phenotype, so we named the mutant as *Bnclavata-like* (*Bnclv-like*). Phenotype analysis illustrated that abnormal development of the inflorescence meristem (IM) led to the fused-inflorescence phenotype. At the stage of protein abundance, major regulators in metabolic processes, ROS metabolism, and cytoskeleton formation were seen to be altered in this mutant. These results not only revealed the relationship between biological processes and inflorescence meristem development, but also suggest bioengineering strategies for the improved breeding and production of *Brassica napus*.

## Introduction

As one of the four greatest oil crops in the world, *Brassica napus* L., plays a crucial role in world oil crops. First of all, rapeseed is an essential organic material for edible oil, and it is rich in fatty acids (such as linoleic acid, linolenic acid). Secondly, rapeseed meal is rich in protein, which is a potential source for the feed protein. Meanwhile, rapeseed stalks, like wheat and maize, can also be used as raw materials for the production of new bio-energy and as an important energy crop. Rapeseed is also a great source of nectar and ornamental plants (Wang *et al.*, 2016). Because the three components of inflorescence structure (number of siliques per plant, number of seeds per silique and 1000-seed weight) is closely correlated with the seed yield in rapeseed, the discovery of optimal inflorescence structure will be helpful to improve the production of rapeseed (Chen *et al.*, 2007; Lu *et al.*, 2017; Zhang *et al.*, 2018).

Inflorescence development affects plant morphogenesis, yield and quality. Studies in *Arabidopsis thaliana* and rice have demonstrated that transcription factors and hormones play a significant role in inflorescence development and lateral branching regulation (Hofmann, 2009; Bongers *et al.*, 2014; Chew *et al.*, 2014; Leduc *et al.*, 2014; Cai *et al.*, 2016; Li *et al.*, 2017; Wang and Jiao, 2018). In *Arabidopsis thaliana*, *TERNIMAL FLOWER 1* (*TFL1*), *LEAFY* (*LFY*) and *APALA 1* (*AP1*) are characteristic genes of the floral meristem, and their antagonistic interac tions can regulate inflorescence branching patterns (Ma *et al.*, 2017). *TFL1* was specifically expressed in main inflorescence meristem and lateral inflorescence meristem, while *LFY* and *AP1* were abundantly asserted in the floral meristem (Winter *et al.*, 2015). The TFL1 loss-of-function mutant may cause heterotopic expression of *LFY* and *AP1* genes, contributing to the transformation of inflorescence meristem into floral meristem, precocious flowering formless inflorescence branching in *Arabidopsis thaliana*. On the contrary, overexpression of *TFL1* in *Arabidopsis thaliana* could inhibit the expression of *LFY* and *AP1*, and thus delay flowering and increase inflorescence branching (Cheng *et al.*, 2018). AP1 protein and its homologs CAULIFLOWER (CAL) and FRUITFULL (FUL) in *Arabidopsis thaliana* could inhibit the expression of *TFL1* gene (Parcy *et al.*, 2002), while LFY protein can promote the expression of *TFL1* gene (Serrano-Mislata *et al.*, 2017). *Arabidopsis* ARGONAUTE1 (AGO1) could also inhibit the expression of *TFL1* gene and regulate inflorescence development (Fernandez-Nohales *et al.*, 2014). SHORT VEGETATIVE PHASE (SVP), SUPPRESSOR OF OVEREXPRESSION OF CONSTANS 1 (SOC1), AGAMOUS-LIKE 24 (AGL24) and SEPLLATA 4 (SEP4) belong to MADS-box transcription factors, which could regulate flowering time and directly inhibit the expression of *TFL1* in newly floral meristem, and thus regulate inflorescence development (Liu *et al.*, 2013).

Phytohormones, especially auxin (IAA) and cytokinin (CK), are key regulators of inflorescence structure, playing an important role in inflorescence growth and development (Benkova *et al.*, 2003; Heisler *et al.*, 2005; Shani *et al.*, 2006; Werner and Schmulling, 2009). *AUXIN-RESPONSE FACTOR* (*ARF*) gene could directly induce the expression of *LFY* and two *AP2* homologous genes (*AINTEGUMENTA* and *AIGUMENTA-LIKE6/PLETHORA3*) (Krizek, 2009; Krizek and Eaddy, 2012; Yamaguchi *et al.*, 2013; Krogan *et al.*, 2016; Carey and Krogan, 2017). The *LFY* gene in *Arabidopsis* also participates in feedback regulation of auxin biosynthesis pathway by inhibiting the expression of some auxin biosynthesis genes, such as *YUCCA1* (*YUC1*) and *YUCCA4* (*YUC4*) (Moyroud *et al.*, 2011; Li *et al.*, 2013; Winter *et al.*, 2015). However, *LFY* gene could promote the expression of *PINOID* (*PID*), an auxin transport regulator (Yamaguchi *et al.*, 2013). In *Arabidopsis*, cytokinins promote inflorescence meristem development and affect inflorescence structure by promoting the expression of *WUSCHEL* (*WUS*) gene and inhibiting the expression of *CLAVATA1* (*CLV1*) and *CLV3* (Gordon *et al.*, 2009). *LONELY GUY* (*LOG*), encoding a cytokinin-activating enzyme, catalyzes the last step of CK biosynthesis. There are nine *LOG* homologous genes in *Arabidopsis thaliana*. The *log3*, *log4*, *log7* triple mutant and *log1*, *log2*, *log3*, *log4*, *log5*, *log7*, *log8* seven-mutant produce fewer floral meristem, indicating that the development of inflorescence meristem requires CK (Kuroha *et al.*, 2009; Tokunaga *et al.*, 2012). In *Arabidopsis*, AP1 could reduce the expression level of CK biosynthesis gene *LOG1*, but activate the cytokinin-degrading gene *CKX3* by binding directly to the promoter of the target gene (Ma *et al.*, 2017; Joshi *et al.*, 2018). The study in *Arabidopsis* showed that mutations in *AHK2*, *AHK3* and *AHK4*, which encodes CK receptor histidine kinase, reduced inflorescence stem length (Nishimura *et al.*, 2004).

Optimized inflorescence architecture is fundamental for high-yield breeding of rapeseed. Thus, much research has been done on the genetic mechanism of inflorescence structure (Cai *et al.*, 2016; Zhao *et al.*, 2016; Zhang *et al.*, 2018). However, insufficient information is available on the development of rapeseed. Here, we present *Bnclv-like*, a natural *B. napus* mutant, which was characterized by abnormal development of inflorescence meristem (IM). Two-dimensional electrophoresis (2-DE) was used to reveal the mechanism of the change in protein level. The proteins involved in IM regulation displayed significant variation, which could provid molecular basis for IM development and inflorescence structure formation in *Brassica napus*.

## Materials and Methods

### Plant materials and growth conditions

In this study, *B. napus* plants (*Bnclv-like* and Ningyou 12) were grown in the experimental field of Jiangsu University. The IM samples for proteomic analysis were collected when the first flower was opening, so that the development of IMs from the mutant and the wild type could keep the same stage. All samples were frozen with liquid nitrogen immediately after harvest and stored at -80 ^o^C before use.

### Protein extraction

The total high-quality proteins from *Bnclv-like* mutant and Ningyou 12 (1.5 g [FW]) were extracted using the ReadyPrep protein extraction kit (Bio-Rad, USA) according to the manufacturer’s instruction with some modifications. Protein concentrations were determined using the RCDC Kit (Bio-Rad, USA) according to the manufacturer’s instruction.

### Two-dimensional electrophoresis (2-DE) and image analysis

2-DE was carried out with 17 cm Immobiline DryStrips (Bio-Rad, USA, linear, pH 4-7) as using a modification of the method of Yang (Yang *et al.*, 2014). First, 1,200 μg of total protein was loaded onto the Immobiline DryStrip using passive rehydration (12 h). Second, isoelectric focusing (IEF) was performed on an IPGphor III IEF system (GE Healthcare, USA) with these steps: at 300, 500, 1,000 and 8,000 V for 1 h each and then held at 8,000 V until a total voltage of 54,000 Vh was reached. Third, the isoelectric focused strips were equilibrated for 15 min in equilibration buffer (0.05 M Tris-HCl, pH 6.8, 2.5% SDS, 30% v/v glycerol and 1% DTT) and then equilibrated again for 15 min (0.05 M Tris-HCl, pH 6.8, 2.5% SDS, 30% (v/v) glycerol and 2.5% (w/v) iodoacetamide). Fourth, second-dimensional electrophoresis was done with a Laemmli buffer system using 5% stacking gels and 15% resolving gels. At last, the gels were stained with 0.116% Coomassie brilliant blue R-250 in a solution containing 25% (v/v) ethanol and 8% acetic acid.

The 2-DE gels were scanned by ImageScanner III (GE Healthcare, USA) at transparency mode with 300 dpi resolution. Gel comparison and spot analysis were performed using ImageMaster^TM^ 2D platinum version 7.0 software (GE Healthcare, USA) according to the manufacturer’s instruction. The intensity ratio of the corresponding spots in different gels was calculated and spots with a ratio ≥2 and an ANOVA ≤0.05 were defined as differential spots. The experiment was repeated three times with independent samples.

### Mass spectrometry (MS) analysis and data analysis

The differential protein spots in *Bnclv-like* mutant and Ningyou 12 were excised manually from the gels and rinsed in ultrapure water with two rounds of ultrasonic treatment (10 min/each). The proteins were digested in gels according to the method of Yang *et al.* (2014). Then, the peptides in the resulting digestion were identified by MALDI-TOF MS (Bruker Daltonics, Ultraflex-TOF-TOF, Germany).

The database searching and protein identification of the peptide mass fingerprinting was performed as described by Yao *et al.* (2011). *B. napus* was selected as the taxonomic category. Proteins with a Mascot score > 64 were considered to be credible.

### Gene ontology analysis of differential proteins

The Gene Ontology (GO) IDs of the identified proteins were obtained through InterProscan searching with the amino acid sequences and were output in txt format. Subsequently, the annotation files of up- and down-regulated proteins and unique proteins in *Bnclv-like* mutant and Ningyou 12 were respectively uploaded in InterproScan.txt into WEGO (Ye *et al.*, 2006; Ye *et al.*, 2018). Finally, the analysis results were output as a histogram file after online operation. The protein-protein interaction network was initially constructed from differential proteins using the STRING database and reconstructed by Cytoscape.

### RNA extraction and quantitative real-time PCR

To validate the differential proteins, quantitative real-time PCR (qPCR) was used to confirm the expression patterns of selected proteins in *Bnclv-like* and Ningyou12. The total RNA of collected samples were extracted using TRIzol reagent (Life technologies, USA) following the protocol of the supplier. First strand cDNA was synthesized by reverse transcription of total RNA (500 ng) using the HiScript Q RT SuperMix for qPCR kit (Vazyme, China). All reactions were performed with an ABI 7300 Real-Time PCR Detection System (Applied Biosystems, USA) with SYBR Green Master Mix (Vazyme, China). Primer premier 5.0 was used to design gene-specific primers according to the corresponding unigene sequences. The sequences of primers were listed in Table S1. Primers were checked for efficiency using the standard curve method, and their specificities were checked using melting curves after all qPCR runs. All qPCRs were performed in triplicate in a total volume of 20 μL. The *ACTIN* gene was used as an internal reference gene. The relative expression levels of genes were calculated using the 2^-ᐃᐃCt^ method.

## Results

### Morphological and genetic characterizations of *Bnclv-like* mutant

**Figure 1:**
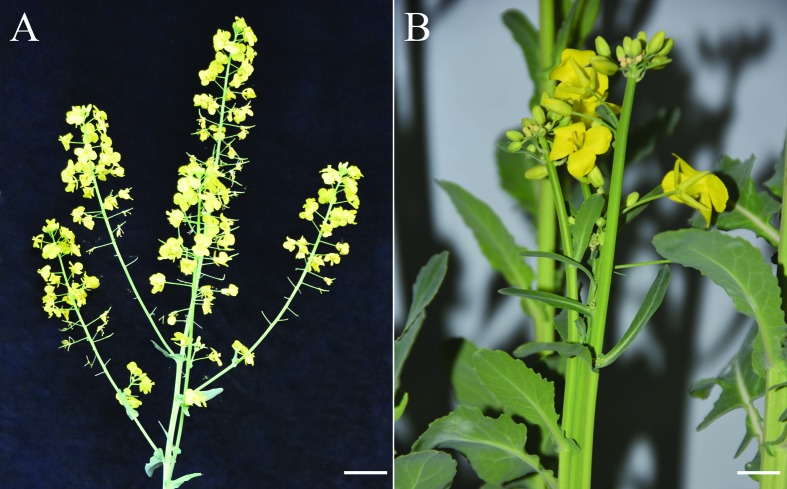
Inflorescence morphology of wild type (A) and Bnclv-like mutant (B) at flowering stage. Bar=10 cm.

We obtained a natural mutant in Ningyou 12 experimental field, which showed fused-inflorescence branching at the flowering stage (Figure 1), similar to the *Arabidopsis* clavata-like phenotype (Brand *et al.*, 2000; Liu *et al.*, 2009), therefore we named the mutant as *Bnclavata-like* (*Bnclv-like*). The *Bnclv-like* homozygote was obtained through self-crossing for five generations, which showed stable inheritance with no segregation of phenotypic traits was observed. Like the *Bnclv-like* mutant, the F_1_ of hybrid between *Bnclv-like* mutant and ZS11 (Zhongshuang 11) also exhibited the fused-inflorescence phenotype. Among 42 F_2_ individuals, 32 and 10 plants were identified as *Bnclv-like* mutant and wild-type, respectively, which fitted an expected Mendelian segregation ratio of 3:1 (χ2=0.02, *P*=0.90). These results indicated that *Bnclv-like* mutant was controlled by a dominant gene.

### Protein expression profiles and differential proteins between *Bnclv-like* mutant and ZS11 in IM

**Figure 2:**
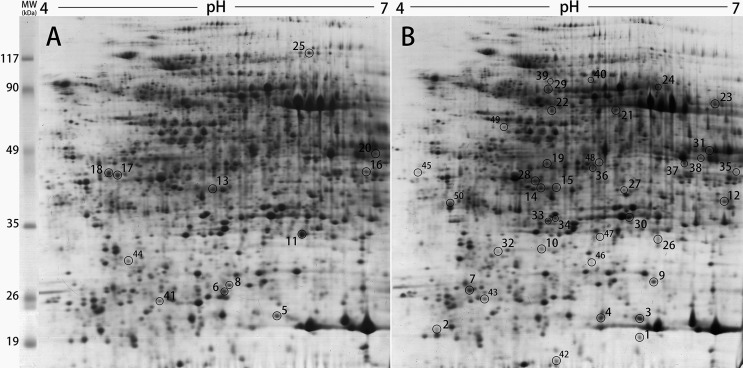
The proteomic profiles of wild type (A) and Bnclv-like (B). The proteins which are upregulated or expressed de novo in Bnclv-like are marked in (B) and downregulated proteins are marked in (A). The numbers indicated represent the match ID of the proteins analyzed by ImageMaster7.0 and listed in Table 1.

Proteomic analysis has been widely used in the identification of various proteins in plants (Yang *et al.*, 2014; Zhu *et al.*, 2014; Wu *et al.*, 2015; Yang *et al.*, 2015; Apaliya *et al.*, 2019). In this study, 17 cm Immobiline DryStrips (pH 4-7, linear) were used for 2-DE analysis. More than 1200 reproducible protein spots were detected in 2-DE gels (Figure 2). Fifty spots were detected to be significantly differentially expressed (ANOVA ≤0.05) (Figure 2). Relative to the wild type, 25 proteins were up-regulated and 12 proteins down-regulated in the *Bnclv-like* mutant. We also found 13 unique proteins in the *Bnclv-like*, indicating that the *Bnclv-like* mutation induces *de novo* accumulation of these proteins.

### Protein identification by MALDI-TOF-MS and functional classification

**Figure 3:**
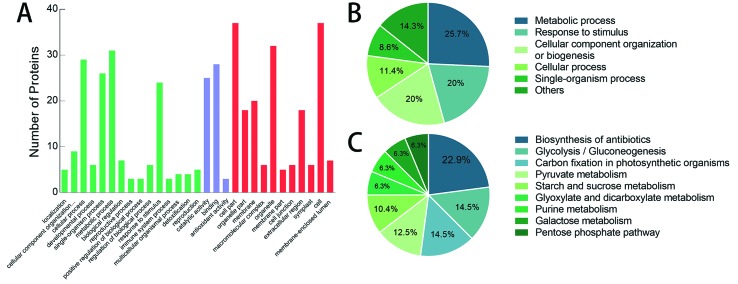
Annotation and classification of differential proteins according to GO and KEGG pathway analysis. (A) Classification of significantly differential proteins annotated by Blast2GO. (B) Reclassification of the biological processes annotated in (A). (C) KEGG pathway analysis of the differential proteins annotated through Blast2GO.

After MALDI-TOF-MS analysis, 41 spots were identified successfully (Table 1). To further predict and classify the function of these proteins, the sequences of these differential proteins were used to search for genes with GO assignments. Of the 41 proteins, 37 were annotated successfully and classified to the categories of molecular function, biological process and cellular component (Figure 3A). Fifteen functional sub-categories were identified for biological process, 11 for the cellular component and 3 for molecular function. Some of the proteins were assigned to more than one sub-category. Therefore, based on the biological function of these proteins, we performed an accurate classification of the biological process (Figure 3B). The largest three sub-categories were “metabolic process”, “response to stimulus” and “cellular component organization or biogenesis”, which were essentially consistent with the results generated by BLAST2GO.

**Table 1:** Identification of differentially expressed proteins between *Bnclv-like* and ZS11 in IM.

Spot No.	NCBI Accession No.	Description	Homolog in *A.thaliana*	MW (kDa)	pI	No.of Amino Acids	No. of Peptide matched	Seq Cover (%)	Score	Fold change
1	gi|923919510	Chlorophyll a-b binding protein 1	ALB3	28.4	5.33	267	12	32	375	2.37↑
2	gi|674896987	Profilin-1	PRF1	14.1	4.48	131	6	29	125	2.71↑
3	gi|674872456	Nucleoside diphosphate kinase 1	NDPK1	15.6	5.91	140	8	26	176	2.64↑
4	gi|674939853	BnaA06g05150D	15.3	5.44	152	8	40	264	3.91↑	
5	gi|674865899	Adenylylsulfatase HINT1	HIT3	14.2	5.91	129	14	74	480	2.1↓
6	gi|923785213	40S ribosomal protein S12-2	Rsp12	15.9	5.54	146	8	36	244	2.08↓
7	gi|923702651	PLAT domain-containing 2	PLAT2	20.5	5.15	182	14	33	430	2.68↑
8	gi|40805177	Eukaryotic translation initiation factor-5A-2	FBR12	17.4	5.56	159	6	25	237	2.81↓
9	gi|923899255	Superoxide dismutase [Cu-Zn]	SOD2	21.5	6.79	207	8	40	458	5.66↑
10	gi|923927649	Elongation factor P (EF-P) family	EF-P	25.8	6.74	230	8	16	193	2.86↑
11	gi|923641432	Fe superoxide dismutase 1	FSD1	23.8	6.16	212	8	11	162	7.59↓
12	gi|674881149	BnaCnng20260D	12.6	4.69	110	12	50	289	3.08↑	
13	gi|674869529	NAD(P)-binding Rossmann-fold superfamily	34.9	8.45	324	10	19	261	4.02↓	
14	gi|674929671	BnaA02g15190D	25.4	5.19	229	9	28	248	3.45↑	
15	gi|674901017	Actin-7	ACTIN 7	39.3	5.2	353	18	32	476	9.34↑
16	gi|923719605	Ferredoxin-NADP(+)- oxidoreductase 1	FNR1	42.7	8.29	378	18	33	368	2.4↓
17	gi|923625827	Glucan endo-1,3-beta- acidic isoform	BG2	37.7	4.78	340	18	26	352	2.93↓
18	gi|674961653	BnaC08g28150D	36.4	4.7	329	20	35	443	3.42↓	
19	gi|923681807	Glucose-6-phosphate 1-epimerase	34.1	5.98	306	11	34	209	2.49↑	
20	gi|937575704	Glyceraldehyde-3-phosphate cytosolic	GAPC1	37	7.7	339	9	25	306	2.26↓
21	gi|923621706	UDP-glucose pyrophosphorylase	UGP1	51.8	5.41	469	14	20	435	2.2↑
22	gi|100801746	Gamma-glutamylcysteine synthetase	GSH1	58.3	6.02	514	28	24	612	20.9↑
23	gi|674912853	NADP-dependent glyceraldehyde-3-phosphate dehydrogenase	ALDH11A3	54.7	6.43	503	20	22	397	7.11↑
24	gi|674889463	Probable mitochondrial-processing peptidase subuni	MPPBETA	58.9	6.23	529	21	29	536	5.71↑
25	gi|674885646	Hsp70-Hsp90 organizing 2	RING/U-box	63.9	5.77	562	40	43	860	2.34↓
26	gi|923541562	Germin subfamily 3 member 3	GER3	22	6.4	211	6	27	274	3.45↑
27	gi|674868327	Probable 6-phosphogluconolactonase chloroplastic	PGL1	28	5.67	255	11	21	230	7.67↑
28	gi|923846509	Proteasome subunit alpha type-1-A	PAF1	30.4	5.09	277	8	18	326	2.39↑
29	gi|383930459	ATPase alpha subunit	ATPA	55.3	5.14	507	23	29	588	3.15↑
30	gi|923604870	Plastid isoform triose phosphate isomerase	TIM	27.5	5.38	254	28	64	859	7.12↑
31	gi|923902961	Fructose-bisphosphate aldolase 8	FBA8	38.8	6.28	358	29	47	951	2.39↑
32	gi|937575319	2-Cys peroxiredoxin 1	ACHT1	29.7	5.81	270	8	17	258	
33	gi|923744137	20 kDa chloroplastic chaperonin 20	CPN20	26.4	8.57	250	20	62	602	
34	gi|923604874	Triosephosphate isomerase	TPI	27.5	5.84	254	9	26	177	
35	gi|937575958	Malate dehydrogenase	MMDH1	35.9	8.81	341	10	19	277	
36	gi|937575063	Annexin D1	ANN1	36.3	5.34	317	10	23	157	
37	gi|923879591	NADP-dependent D-sorbitol-6-phosphate dehydrogenase	S6PDH	35.3	6.02	309	10	22	232	
38	gi|923708436	Cytosolic malate dehydrogenase	C-NAD-MDH1	35.8	6.11	332	15	31	220	
39	gi|923709448	ATP binding cassette protein 1	ABCI8	61.8	5.75	552	8	8	108	
40	gi|923539571	NADP-dependent malic enzyme 2	NADP-ME2	64.9	5.3	588	8	7	101	
41	gi|923878119	Glycine-rich RNA-binding GRP1A isoform X1	GRP1A	16.2	5.5	167	4	19	140	4.66↓

The information about metabolic pathways of the differential proteins is valuable for identifying altered physiological processes in the *Bnclv-like* IM. KEGG pathway analysis was performed subsequently. Twenty-one out of 37 annotated proteins were mapped to 41 biological pathways, among which “biosynthesis of antibiotics”, “glycolysis/gluconeogenesis” and “carbon fixation in photosynthetic organisms” were the three largest pathways, consisting of 11, 7 and 7 proteins, respectively (Figure 3C).

**Figure 4:**
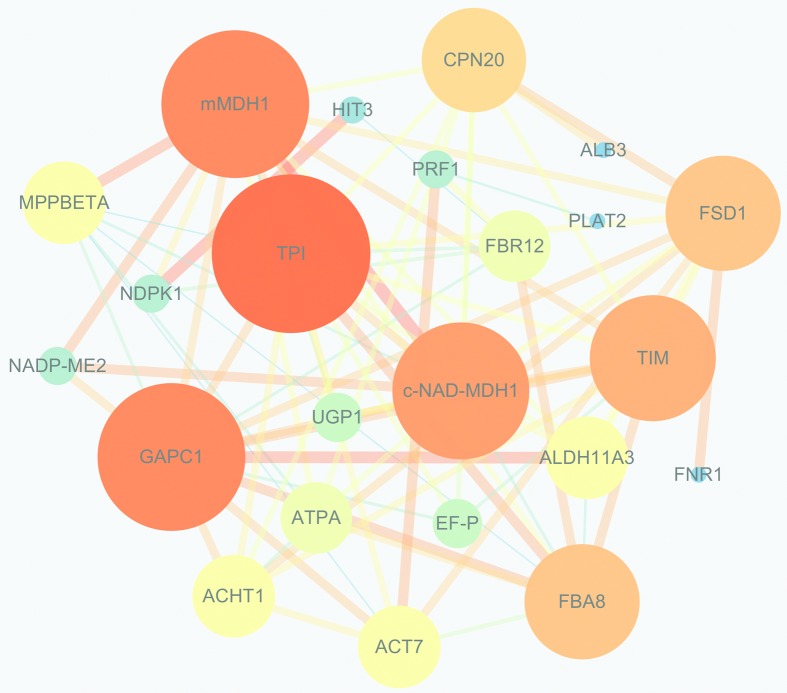
Schematic representation of the protein-protein interaction network of the differentlial proteins in the Bnclv-like. Proteins with larger numbers of interacting proteins are represented by a larger circle size and color depth. The line width represents the reliability of the predicted interaction between two proteins, where interactions containing more evidence are thicker. The network was initially constructed from Bnclv-like differential proteins using the STRING database and reconstructed by Cytoscape.

**Figure 5:**
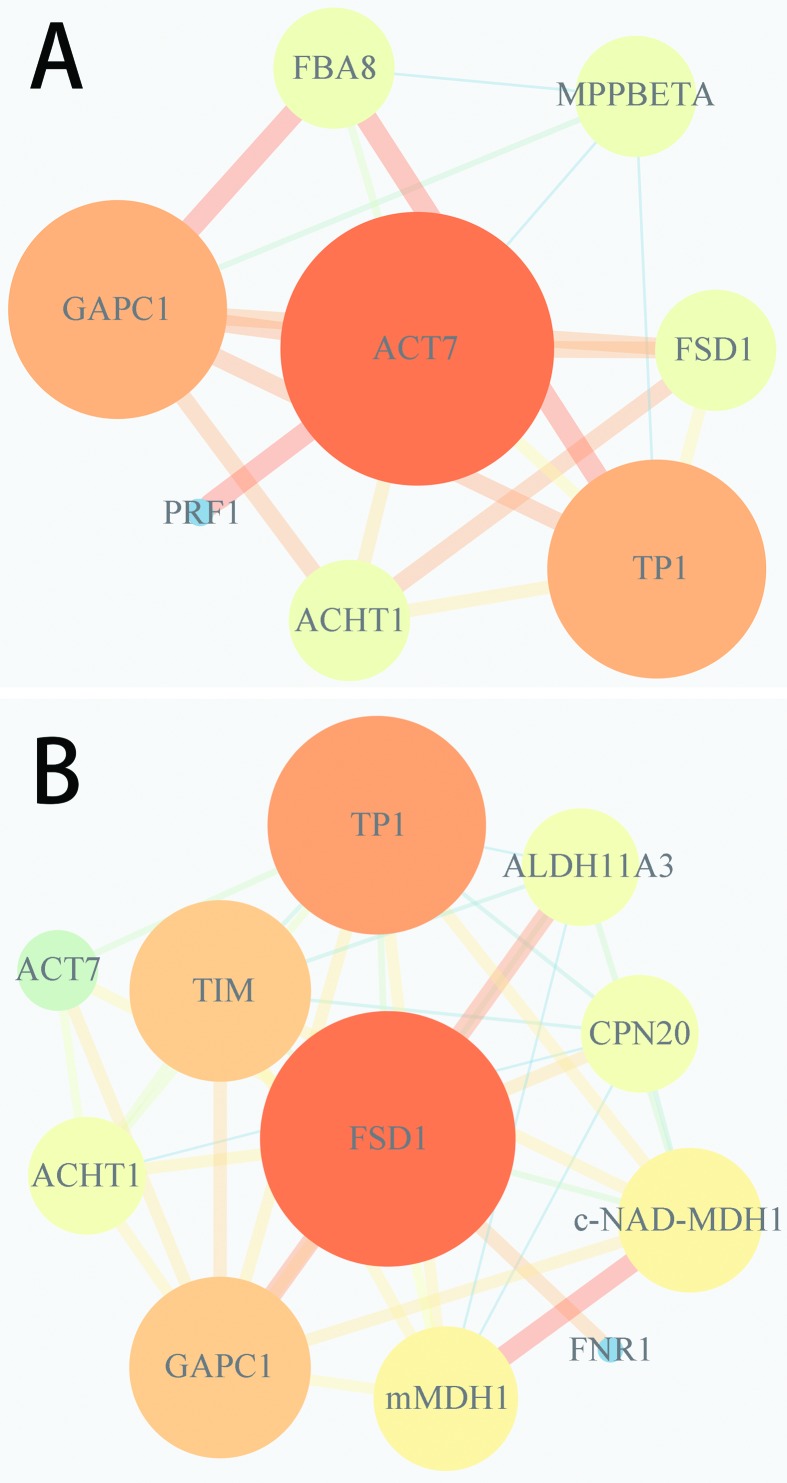
Schematic representation of the protein-protein interaction network of the differential proteins in the bnclv-like that interact with act7 (a) and fsd1 (b), respectively. proteins with larger numbers of interacting proteins are represented by a larger circle size and color depth. The line width represents the reliability of the predicted interaction between two proteins, where interactions containing more evidence are thicker. The network was initially constructed from Bnclv-like differential proteins using the STRING database and reconstructed by Cytoscape.

To further investigate the roles of differential proteins in the abnormal IM development in the *Bnclv-like* mutant, we searched for evidence of direct or functional protein-protein interactions (PPI). Based on their GO annotations, 37 proteins were chosen for PPI analysis. The results showed that 23 of them were predicted to interact with each other (Figure 4). In the network, TPI, GAPC1, c-NAD-MDH1, and mMDH1 were predicted to have the most interactions with other proteins. The up-regulation of TPI, c-NAD-MDH1, and mMDH1 might be a central contribution to the development of *Bnclv-like* IM. In addition to proteins related to metabolism, the interaction network also contained proteins involved in cytoskeleton construction and stress responses. ACT7 was up-regulated 9-fold and the expression of FSD1 was down-regulated 7-fold. In order to reveal how cytoskeletal formation and stress response proteins are related with the abnormal IM development in *Bnclv-like*, these two proteins were selected as the center of these two pathways to analyze the interacting networks around them. The results showed that seven proteins interacted with ACT7 (Figure 5A) and 10 proteins interacted with FSD1 (Figure 5B). Interestingly, three proteins showed interactions with both ACT7 and FSD1, indicating a connection between these two biological processes.

### Quantitative real-time PCR

**Figure 6:**
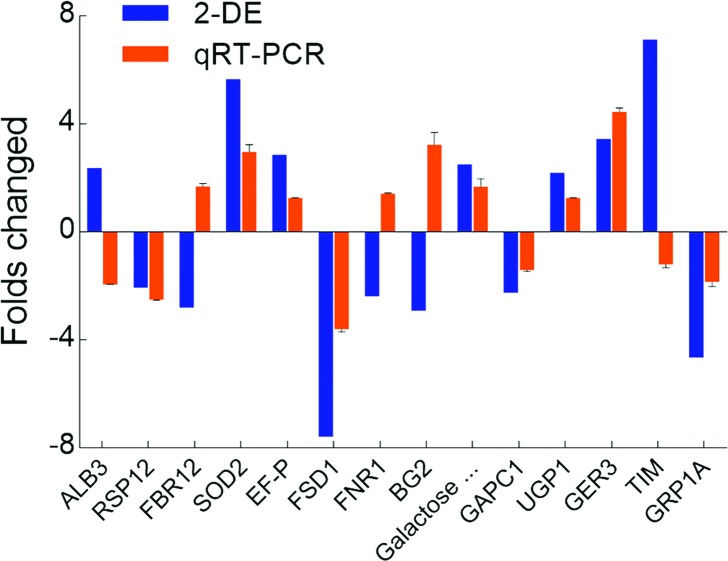
Comparison of the results obtained from 2-DE with those from qPCR. The results obtained by 2-DE and qPCR are marked in blue and orange columns, respectively. The Y-axis represents the fold-change in the Bnclv-like mutant relative to the wild type.

To confirm the accuracy of the 2-DE results, 19 genes were selected for qPCR validation (Figure S1). Fourteen genes displayed the same trend variations with the results of 2-DE, whereas five genes exhibited different directions of change in expression (Figure 6). Surprisingly, the proteins found to be expressed only in the *Bnclv-like* background were detected in the wild type using qPCR, such as CPN20, mMDH1 and S6PDH (Figure S1). This may be due to a post-transcriptional modification of mRNAs. Collectively, at the protein level, three biological processes made major contributions to the abnormal development of the IM in *Bnclv-like*. The up-regulation of proteins in the metabolic processes and cytoskeleton formation could provide enough energy and faster transportation of cellular materials for fulfilling the higher activity of the *Bnclv-like* in IM. On the other hand, the downregulation of proteins involved in ROS metabolism might have a positive influence on the maintenance of stem cell activity. In general, the qRT-PCR results showed that the transcriptional and protein levels of the fourteen proteins were the same.

## Discussion

In present study, we obtained a natural mutant of rapeseed named as *Bnclv-like*, which exhibited abnormal inflorescence formation. We speculated that the *Bnclv-like* phenotype was caused by abnormal development of the IM. So, the proteomic analysis was implemented to further investigate the unusual IM development in *Bnclv-like.* Using the GO classification and KEGG pathway analysis of the differential proteins between the *Bnclv-like* mutant and wid-type IM, we found that these differential proteins were mainly involved in metabolic processes, responses to stimulus and cellular component organization or biogenesis.

Plants need a lot of ATP for energy during the whole growth and development process (Parker *et al.*, 2006; Kang *et al.*, 2012). From KEGG pathway analysis, we identified seven proteins belonging to the glycolysis/gluconeogenesis pathway and seven proteins participating in the process of carbon fixation in photosynthetic organisms. GAPC1, TPI, and TIM are involved in these two pathways simultaneously. GAPC1 (Phosphorylating glyceraldehyde-3-P dehydrogenase) is a highly conserved cytosolic enzyme, but it is also thought to be related to other cellular functions apart from its participation in glycolysis. The *gapc1* mutant exhibits delayed growth, altered silique morphology, and decreased ATP level and respiratory rate (Rius *et al.*, 2008). However, *GAPC1* overexpression had no significant influence on seedlings in the vegetative stage, which presented a seed-specific expression pattern of *GAPC1* (Guo *et al.*, 2014). In plants, triose phosphate isomerase (TPI) participates in several metabolic processes, including gluconeogenesis, glycolysis, and the Calvin cycle. One or various *TPIs* are present in plant genomes and are located in the cytoplasm and chloroplast (cTPI and pdTPI), respectively. cTPI is involved in glycolysis, whereas the chloroplastic enzymes participate in the Calvin cycle (Turner *et al.*, 1965; Kurzok and Feierabend, 1984; Tang *et al.*, 2000; Chen and Thelen, 2010). In *Arabidopsis*, the lack of pdTPI results in termination of the transition from vegetative to reproductive stages or plants suffers from stunted growth and abnormal development of chloroplasts (Lopez-Castillo *et al.*, 2016). In the present study, the expression of TIM and TPI was upregulated, which might contribute to energy metabolism in the IM in the *Bnclv-like* mutant.

Actin plays a key role in regulating organ growth, cell proliferation and floral bud morphogenesis from vegetative to reproductive stages in plants (Feng *et al.*, 2006; Zhang *et al.*, 2013; Zheng *et al.*, 2013; Wu *et al.*, 2016). The *Arabidopsis ACT7* gene is expressed in rapidly developing tissues, in which the highest level of *ACT7* mRNA could be detected in developing vegetative organs (McDowell *et al.*, 1996). In addition, *ACT7* is the only actin gene in *Arabidopsis* that responds strongly to auxin (McDowell *et al.*, 1996). A recent study demonstrated that ACT7 participated in the process of TWISTED DWARF1 (TWD1) mediation of auxin transport. Although ACT7 may be an indirect-TWD1 interactor, it controls the presence of efflux transporters at the plasma membrane. As a consequence, *act7* and *twd1* mutants shared developmental and physiological phenotypes indicative of defects in auxin transport (Zhu *et al.*, 2016). Our data showed that the expression level of ACT7 protein was significantly up-regulated in the *Bnclv-like* mutant. Taken together, the highly expressed ACT7 in *Bnclv-like* mutant might promote cell division and growth during IM development.

ROS are well-known stress responding molecules in plants and animals which can be increased dramatically in response to pathogens and environmental stresses (Finkel and Holbrook, 2000; Swanson and Gilroy, 2010). A recent study indicated that redox participated in the regulation of plant stem cell fate (Zeng *et al.*, 2017). O_2_^-^, the precursor for most ROS, can be transformed into H_2_O_2_ by superoxide dismutase (SODs). Ideal concentrations of O_2_^-^ can stabilize the activity of stem cells, but excess H_2_O_2_ can suppress or even disrupt their activity (Zeng *et al.*, 2017). Two SODs, SOD2, and FSD1, were altered significantly in the *Bnclv-like* IM. In a previous report, these two proteins were found to be strongly expressed in the differentiating peripheral zone instead of the stem cells as a result of the different distribution of O_2_^-^ (Yadav *et al.*, 2014; Zeng *et al.*, 2017). In the present study, the expression of SOD2 was up-regulated dramatically, which could catalyze the transformation from O_2_^-^ to H_2_O_2_ to suppress stem cell activity. However, FSD1 showed a more significant down-regulation than SOD2, which may compensate for the elevated activity of SOD2. Another study indicated that ROS were crucial molecules in triggering meiotic fate acquisition in maize (Kelliher and Walbot, 2012), which demonstrated an important role of ROS in cell fate determination.

The PPI network showed that proteins involved in cell metabolism, cytoskeleton formation and ROS metabolism interact with each other. Due to their crucial role in cytoskeleton formation and ROS metabolism in cell development, ACT7 and FSD1 were selected for further analysis. The number of proteins interacting with ACT7 and FSD1 accounted for > 70% in all interacting with proteins, indicating the vital role of these two processes in regulating the development of the *Bnclv-like* mutant IM.

Among the proteins interacting with ACT7, PRF1 encodes profilin. The *vitro* studies had shown that the profilin-actin complexes were associated with the barbed ends of actin filaments and promoted actin polymerization by reducing the critical concentration and increasing nucleotide exchange on G-actin (Pollard and Cooper, 1984; Pantaloni and Carlier, 1993). In *Arabidopsis thaliana*, PRF1 participates in stochastic actin dynamics by regulating formin-mediated actin nucleation and filament elongation in the process of axial cell expansion (Cao *et al.*, 2016). Consistent with our results, the expression of PRF1 in the *Bnclv-like* mutant is up-regulated relative to the wild type, together with ACT7, which is consistent with the enrichment of ACT7. Fructose 1, 6-biphosphate aldolase (FBA) in plants is a key metabolic enzyme in glycolysis and gluconeogenesis in the cytoplasm (Gross *et al.*, 1999). FBA8 is a member of the cytoplasmic fructose 1, 6-biphosphate aldolase family. A recent study showed that the knockout of the *FBA8* gene resulted in slight alternations of the actin cytoskeleton morphology of guard cell and reduced the rate of stomatal closure in cope with decreased humidity (Garagounis *et al.*, 2017). Moreover, the *fba8* mutant displayed sterility (Lu *et al.*, 2012). *In vitro* experiments confirmed the interaction between FBA8 and actin in *Arabidopsis* (Lu *et al.*, 2012). Due to the significant role in cytoskeleton formation and glucose metabolism, FBA8 may provide a link between these two processes. The up-regulation of PRF1 and FBA8 could enhance the development of IM through their interaction with ACT7.

Among the proteins interacting with FSD1, *Arabidopsis* chloroplast CHAPERONIN 20 (CPN20) can form tetramers *in vitro*, which is a cofactor of chaperonin (Koumoto *et al.*, 1999)*.* In *Arabidopsis*, CPN20 is speculated to have many functions in the chloroplast independent of its co-chaperonin, such as regulating abscisic acid signaling transduction and mediating iron SOD activity (Kuo *et al.*, 2013; Zhang *et al.*, 2014). CPN20 was identified as a mediator for activating FeSOD by direct interaction *in vivo* and *in vitro* (Kuo *et al.*, 2013). mMDH1 encodes a mitochondrial malate dehydrogenase, which participates in the transformation of malic acid and oxaloacetic acid in the tricarboxylic acid cycle. A decreased activity of mMDH1 has a up-regulated influence on photorespiratory metabolism, which leads to smaller rosettes and decreased fresh weight (Linden *et al.*, 2016; Sew *et al.*, 2016). The *mmdh1mmdh2* double mutant plants exhibit a significantly higher rate of leaf respiration, low net CO_2_ assimilation, limitation in photorespiratory rate, and slow-growth phenotypes in rosettes (Tomaz *et al.*, 2010; Linden *et al.*, 2016). In the *Bnclv-like* mutant, the upregulation of CPN20 and mMDH1 contribute to the protein biosynthesis and biomass accumulation to maintain the accelerated activity of IM. Besides, the interaction among CPN20, mMDH1, and FSD1 could represent the transformation from energy metabolism to reactive oxygen metabolism in the plant body. A further study should be undertaken to reveal the relationship between these two processes.

We found that the three proteins, TPI, GAPC1, and ACHT1, showed interactions between with ACT7 and FSD1. The first two of them participate in glycometabolism, while ACHT1 is involved in regulating photosynthetic electron transport progress (Dangoor *et al.*, 2012). Therefore, we proposed that energy metabolism could be a link connecting cell organization and superoxide metabolism. Taken together, in protein level, three biological processes showed a great contribution to the abnormal development of *Bnclv-like* mutant and the understanding of interaction between these proteins could be key to uncover the inner mechanism of IM development. This study provided clues for the further study of the *Bnclv-like* mutant in *B. napus* and the mutant was also a useful material for the study of IM development in *B. napus.*

## Supplementary material

The following online material is available for this article:

Figure S1 - The relative expression levels of selected genes which changed significantly in the 2-DE result.

Table S1 - Primers used for qRT-PCR.
