# Supplementary material for: Proteomic analysis of a clavata-like phenotype mutant in Brassica napus
Source: Genet Mol Biol. 2020 Mar 6;43(1):e20190305. doi: 10.1590/1678-4685-GMB-2019-0305 (PMC7198001; doi:10.1590/1678-4685-GMB-2019-0305)
Supplement: Supplementary file 1 [file 1415-4757-GMB-43-1-e20190305-s2.pdf]

Supplementary Material to "Proteomic analysis of a clavata-like phenotype mutant in *Brassica napus*"

Table S1 - Primers used for qRT-PCR.

Gene	Primer name	Primer sequence (5'to 3')
Actin	Actin-QF	TGTTGCTATCCAGGCTGTTCTTTC
	Actin-QR	GATAGCGTGAGGAAGAGCATAACC
ALB3	ALB3-QF	GGAGGACTTGCTTTACCCAGGA
	ALB3-QR	GGTGACAATAGCCTGGACAAAGAA
RSP12	RSP12-QF	TACTGTTCCAAGTGCCAAGACCC
	RSP12-QR	TGTGCTTCTTGACGATGTTGAGTG
FBR12	FBR12-QF	AGCAGGCTGGTAACATCCGTAA
	FBR12-QR	ACTTTGCGTGACCGTGCTTC
SOD2	SOD2-QF	CGGGTGACCTGGGAAACATTA
	SOD2-QR	ACCACAAAGGCTCTTCCAACAA
EF-P	EF-P-QF	AGCCAATCAGTGACCAGAAGGA
	EF-P-QR	CACCTTTGCCTGGCTTCACA
FSD1	FSD1-QF	TACAACAACGGCGACCTCCTC
	FSD1-QR	CGGCAGCAGCATTGAACTCA
FNR1	FNR1-QF	GAGCCTTACACGGCAAAGATAGTT
	FNR1-QR	AATAAAGGCGAACATTGTGAGGAG
BG2	BG2-QF	CGGTCGGTAACGAGGTGAAAC
	BG2-QR	TGTACTCATCCGTGAATCTACCGTG
Glucose-6-phosphat 1 epimerase	G6P-QF	TCTCCAAATCATCTCCGAACCAG
	G6P-QR	TCGGGAGAAGTGAGGACGAGTT
GAPC1	GAPC1-QF	CCACCGTTGATGTTTCAGTTGTT
	GAPC1-QR	CAACATCATCCTCGGTGTAACCA
UGP1	UGP1-QF	TCCAGACGCCCACTGATGAA
	UGP1-QR	TGTCGTCCCAAGACCTCCATT
GER3	GER3-QF	GCGCTGGATTTATCTCATCTGCT
	GER3-QR	CAGGACCCTTCCCAGAGTTGAG
TIM	TIM-QF	CGGAACTGCTGAGGAAGTGAAG
	TIM-QR	GTGCCGCAACATAGAAGTCAGG
CPN20	CPN20-QF	GCTGCTTCTGTGGTTGCTCCTAA
	CPN20QR	CCTTCACCCACAGCAACGACTT
MMDH1	MMDH1-QF	GAAAGGCTAATGTCCCTGTTGC
	MMDH1-QR	CCCTGCTTTAGCCTCCACAAC
S6PDH	S6PDH-QF	TCTCAGAAGCATTCAAGGAAGGTC
	S6PDH-QR	GGGAAATGAACGAGGTAAAGGTC
ABCI8	ABCI8-QF	GAGGAACTGTTACCGTGGTCTTGT
	ABCI8-QR	GCTCAACTTTCGCTGATGGATT
NADP-ME2	NADP-ME2-QF	GATGTGGGTACAAACAACCAGAAGT
	NADP-ME2-QR	CAGAAGGTCAAACGCATTGTGGT
GRP1A	GRP1A-QF	GGGGTTCGGATTCGTCACTT
	GRP1A-QR	GCCTCGTTCACAGTGATGCTG
