# Supplementary material for: Proteomic analysis of a clavata-like phenotype mutant in Brassica napus
Source: Genet Mol Biol. 2020 Mar 6;43(1):e20190305. doi: 10.1590/1678-4685-GMB-2019-0305 (PMC7198001; doi:10.1590/1678-4685-GMB-2019-0305)
Supplement: Supplementary file 2 [file 1415-4757-GMB-43-1-e20190305-s1.pdf]

Supplementary Material to "Proteomic analysis of a clavata-like phenotype mutant in *Brassica napus*"

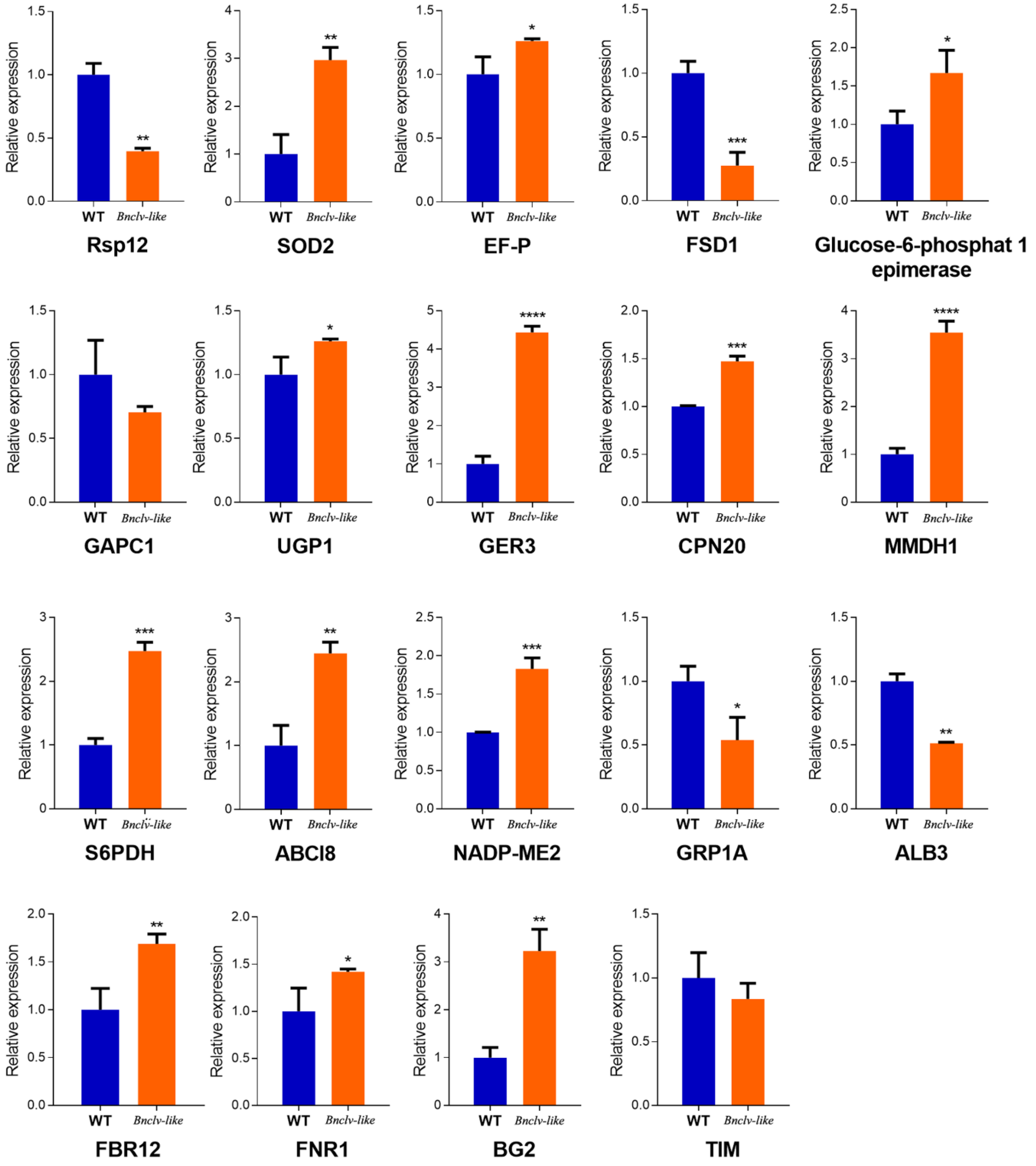

Figure S1 - The relative expression levels of selected genes which changed significantly in the 2-DE result. Y-axis indicates the relative expression level of *Bnclv-like* mutant (orange) compared to wild type (blue). Error bars represent ± standard deviation from 3 biological replicates. *, **, ***, **** indicate significant difference with the wild type at $P<0.05$, 0.01, 0.001 and 0.0001 respectively (*t*-test).
